# Clinical Decision Support System Based on Hybrid Knowledge Modeling: A Case Study of Chronic Kidney Disease-Mineral and Bone Disorder Treatment

**DOI:** 10.3390/ijerph19010226

**Published:** 2021-12-26

**Authors:** Syed Imran Ali, Su Woong Jung, Hafiz Syed Muhammad Bilal, Sang-Ho Lee, Jamil Hussain, Muhammad Afzal, Maqbool Hussain, Taqdir Ali, Taechoong Chung, Sungyoung Lee

**Affiliations:** 1 Department of Computer Science and Engineering, Kyung Hee University, Yongin-si 17104, Korea; imran.ali@oslab.khu.ac.kr (S.I.A.); bilalrizvi@oslab.khu.ac.kr (H.S.M.B.); 2 Department of Internal Medicine, Division of Nephrology, Kyung Hee University Hospital at Gangdong, Seoul 05278, Korea; lshkidney@khu.ac.kr; 3 Department of Computing, SEECS, NUST University, Islamabad 44000, Pakistan; 4 Department of Data Science, Sejong University, Seoul 30019, Korea; jamil@sejong.ac.kr; 5 Department of Software, Sejong University, Seoul 30019, Korea; mafzal@sejong.ac.kr (M.A.); maqbool.hussain@sejong.ac.kr (M.H.); 6 BC Children’s Hospital, University of British Columbia, Vancouver, BC V6H 3N1, Canada; taqdir.ali@bcchr.ca

**Keywords:** clinical decision support system, treatment recommendation, case-based reasoning, medication dosing estimation, expert knowledge modeling

## Abstract

Clinical decision support systems (CDSSs) represent the latest technological transformation in healthcare for assisting clinicians in complex decision-making. Several CDSSs are proposed to deal with a range of clinical tasks such as disease diagnosis, prescription management, and medication ordering. Although a small number of CDSSs have focused on treatment selection, areas such as medication selection and dosing selection remained under-researched. In this regard, this study represents one of the first studies in which a CDSS is proposed for clinicians who manage patients with end-stage renal disease undergoing maintenance hemodialysis, almost all of whom have some manifestation of chronic kidney disease–mineral and bone disorder (CKD–MBD). The primary objective of the system is to aid clinicians in dosage prescription by levering medical domain knowledge as well existing practices. The proposed CDSS is evaluated with a real-world hemodialysis patient dataset acquired from Kyung Hee University Hospital, South Korea. Our evaluation demonstrates overall high compliance based on the concordance metric between the proposed CKD–MBD CDSS recommendations and the routine clinical practice. The concordance rate of overall medication dosing selection is 78.27%. Furthermore, the usability aspects of the system are also evaluated through the User Experience Questionnaire method to highlight the appealing aspects of the system for clinicians. The overall user experience dimension scores for pragmatic, hedonic, and attractiveness are 1.53, 1.48, and 1.41, respectively. A service reliability for the Cronbach’s alpha coefficient greater than 0.7 is achieved using the proposed system, whereas a dependability coefficient of the value 0.84 reveals a significant effect.

## 1. Introduction

Clinical decision support systems (CDSSs) play an important role in enhancing the overall capabilities of healthcare providers [1,2]. In a rapidly changing healthcare landscape, CDSSs are emerging as inevitable applications for informed decision-making. CDSSs are software application systems that provide time-critical, valuable, and relevant information to doctors, paramedical staff, and patients in order to help them deal with complex medical cases. CDSSs are sophisticated systems that encompass a variety of tools, such as alerts/reminders to the caregivers, knowledge extraction from clinical guidelines, medication and test ordering, diagnosis automation, and prescription management [3].

CDSSs provides assistance to medical practitioners in critical decision-making pertaining to patients’ medical situations. The adoption of computerized systems in healthcare has a positive impact on the quality of patient care. In this regard, CDSSs provide medical practitioners with the necessary support in terms of pointers to relevant domain knowledge, highlighting relevant patient information, and decision support to deal with complex situations requiring expert intervention [4]. Although most CDSSs are geared towards medical professionals, some systems also provide support to the patients in terms of education and awareness regarding their medical condition. Before proceeding to the contents, readers are encouraged to look at Table 1 for the abbreviations used in this manuscript.

It is widely indicated in the literature that CDSSs can positively impact the overall quality of healthcare by leveraging state-of-the-art technologies which result in effective and efficient decision management without hindering the established clinical/healthcare workflows. In this regard, it is of the utmost importance that CDSSs provide services without becoming overtly bothersome to the clinicians [5,6]. Therefore, the usability aspects of CDSSs are also an important consideration. The application of these systems can be justified on the basis of their impact on the following: increased quality of service, reliable and transparent decision support, real-time situational awareness, enhanced health outcomes, user satisfaction e.g., of healthcare personnel and/or patients, and time-saving [7].

In this study, we designed a CDSS to assist the management of chronic kidney disease–mineral and bone disorder (CKD–MBD) in patients undergoing maintenance hemodialysis. The kidneys keep blood levels of electrolytes including calcium and phosphate within the normal range by finely handling their urinary excretion. Dysregulation of serum calcium, phosphate, and parathyroid hormone (PTH) begins far before reaching end-stage renal disease, even when kidney function is declined by half [8]. Importantly, biochemical abnormalities are closely interrelated to altered bone turnover and mineralization, and vascular calcification, leading to fractures [9,10] and cardiovascular disease [11], both of which are serious and highly prevalent morbidities in dialysis patients. CKD–MBD is an insidious pathological process complicated in CKD that encompasses biochemical abnormalities, bone abnormalities, and vascular calcification.

**Figure 1:**
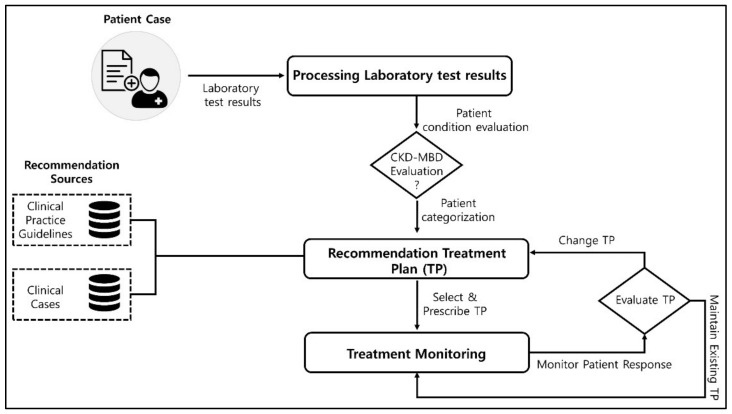
A generic process flow for the chronic kidney disease–mineral and bone disorder (CKD–MBD) treatment regimen selection.

Kidney Disease: Improving Global Outcomes (KDIGO) guidelines recommend serial assessments of serum phosphorus, calcium, and PTH and present their target ranges [12]. However, these three key laboratory values are hard to control within the target range at the same time despite technical advancements in dialysis-related apparatus and the introduction of new medications. Indeed, more than half of patients on dialysis do not achieve recommended target ranges of serum phosphorus, calcium, and PTH levels [13]. Since maintaining serum phosphate, calcium, and PTH within target ranges may reduce cardiovascular events and mortality [14,15] in ESRD patients, their optimal control is of paramount importance. One of the major barriers to correct laboratory abnormalities is that the medication prescribed to control one parameter may cause other parameters to fall out of the target range. To help clinicians prescribe the best set of medications, we developed a computerized decision support system which provides recommendations mostly regarding medication adjustment based on domain knowledge and past patient cases. A generic process flow for the CKD–MBD evaluation and treatment is presented in Figure 1.

As CKD–MBD is not a single disease entity but encompasses a variety of altered mineral and bone metabolisms, a patient is initially evaluated for CKD–MBD by measuring serum calcium, phosphate, and PTH levels, and examining ectopic calcification with lateral abdominal radiography and echocardiography. Then, considering CKD–MBD status and associated clinical situations, an appropriate treatment plan consisting of dietary modification and medications is established. The objective of the proposed CKD–MBD CDSS is to assist clinicians in the selection of an appropriate treatment regimen, i.e., medication selection and dosage recommendations for the management of CKD–MBD in ESRD patients. In this regard, we have focused on the expert-in-the-loop approach, i.e., clinicians provide essential domain knowledge for decision modeling and recommendation generation.

The key contributions of the paper are as follows:

- Active case selection based on domain knowledge;
- Case selection based on reference cases identified through a patient improvement indicator (PII);
- Case adaptation based on domain knowledge and statistical dispersion;
- The proposed system is validated on real-world clinical cases;
- The evaluation of usability aspects to demonstrate higher user experience utility for clinicians.

In order to address the aforementioned points, the study is designed in a collaborative manner in which both domain experts (i.e., clinicians) and knowledge engineers work in tandem to realize the proposed CDSS. The domain experts provide the relevant domain knowledge, which is in turn modeled and enhanced by knowledge engineers. Most of the CDSSs in the domain of CKD are based on black-box machine learning models [16,17,18]. Systems built on such models are generally applied for diagnostic applications where the system provides a prediction along with a confidence score [19,20]. Although black-box models generally exhibit higher accuracy, such data-driven models have limited utility due to sparsity of data, such as in the case of medication intake where a small subset of medications are prescribed more frequently than the others. Therefore, in this paper, we have focused on hybrid knowledge modeling to combine expert knowledge with that of clinical cases of patients for decision support in medication recommendations.

**Figure 2:**
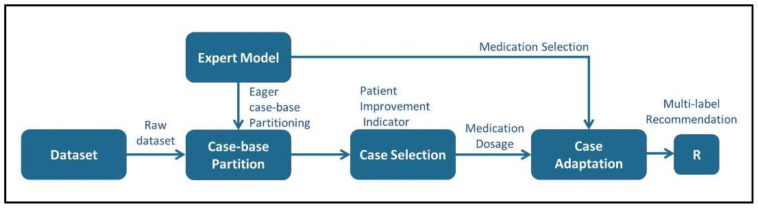
Abstract diagram depicting the role of domain knowledge and clinical cases in the proposed approach.

The abstract idea of the proposed case-based hybridization approach is depicted in Figure 2. The proposed approach is comprised of three major operations, i.e., case-base partitioning, case selection, and case adaptation. The major emphasis of the proposed approach is to synthesize abstract domain knowledge with specific domain cases in order to generate a comprehensive recommendation for a complex scenario.

The main contribution of the study is a proposed hybrid methodology that combines both explicit knowledge (i.e., acquired from domain experts in the form of a partial domain model) and implicit knowledge (i.e., in the form of clinical cases) for complex multi-factor recommendations. Therefore, the medication and dosing selection for CKD–MBD patients is adopted as a case study.

The proposed approach is based on the case-based reasoning (CBR) framework, which imitates a clinician’s thinking and attempts to solve new problems by reusing solutions that have been used to address similar problems in the past. CBR works with specific cases from past scenarios and adapts the outcomes and experiences to an unseen problem. The greater interpretability of the recommendation is a key benefit of the CBR framework. Therefore, clinicians can easily evaluate the CDSS recommendation and follow the line of reasoning followed by the system.

Major differences in this case from a conventional CBR are the development of a domain model and the leveraging of it for case-base partitioning. The case selection is further refined using reference case selection using a Patient Improvement Indicator (PII). A hybrid approach is used for case adaptation using domain based rules and statistical techniques, such as interquartile range. The domain model only partially captures the solution component (i.e., only medication selection is covered by the domain model), we therefore demonstrate how to utilize the partial domain model in conjunction with clinical cases for medication dosage selection. In this regard, both the domain model and clinical cases are employed in a complementary manner through the hybridization pipeline proposed in this paper. The proposed pipeline can be applied to any other medical treatment domains that include medication prescription and dosing adjustment.

## 2. Related Work

Medication management is a tedious and error-prone task for both clinicians and patients. Deep learning-based approaches are generally employed for processing unstructured data, such as medication images and clinical texts, for the purpose of correctly identifying medication information. To reduce medication identification errors by the patients, deep learning-based techniques are leveraged that aid in prescription pill identification from mobile images [21,22,23,24,25]. Similarly, deep learning techniques are also successfully applied to the task of medication and dosage extraction from clinical texts, such as clinical notes [26,27,28] and social media texts [29,30,31]. Moreover, some studies have explored deep learning applications for medication selection focusing on drug–drug interaction [32], dosage selection from free clinical text processing published literature [33], and electronic health records [34], selecting discharge medications based on patient information documented in admission notes [35], among other sources. The aforementioned approaches benefited from training models on a huge amount of data and/or leveraging pre-trained models already available for similar tasks. However, one major hurdle that limits the application of black-box models in clinical practice is the lack of the interpretability of these approaches [36,37,38]. Alternatively, the proposed hybrid case-based approach provides interpretable medication selection and dosage adjustment recommendations given the small amount of clinical data with reasonably acceptable accuracy.

Therefore, in this section, we focus on those aspects of the CDSS that are within the scope of the proposed methodology, such as expert knowledge acquisition, medication selection and dosing adjustment.

### 2.1. Knowledge Acquisition for CDSS Development

A mind-map-based knowledge acquisition process is proposed by Yu et al. [39] for the treatment of thyroid nodules. The authors proposed a consultative process between domain experts and knowledge engineers in which a domain model is produced. A number of clinical practice guidelines (CPGs) pertaining to the thyroid nodule treatment are analyzed by the domain experts and, subsequently, an iterative decision tree (DT) model is generated by the knowledge engineers for automating the decision-making process. The CDSS was evaluated using retrospective medical records of 483 patients. The authors reported 78.9% concordance between the CDSS recommendations and routine clinical practice.

A similar modeling approach is adapted by Choi et al. [40], in which a CDSS for heart failure diagnosis is proposed. The authors proposed a hybrid knowledge modeling approach in which both expert-driven and data-driven models are consolidated into a single model. In this regard, the Classification and Regression Tree (CART) model is used to build a decision tree from patients’ medical records. Moreover, the resulting model is combined with the DT model produced by the domain expert. The authors reported higher accuracy of the combined model as compared with both the expert-driven model and the data-driven model.

Hussain et al. [41] proposed a knowledge validation and verification approach for such cases when multiple stakeholders are involved in the knowledge modeling process and diverse sources are consulted. A hybrid approach was used which consists of both the domain expert knowledge as well as patients’ medical records. The resulting CDSS is used for the treatment of oral cavity cancer patients. It was observed that knowledge verification is an important aspect of expert-based knowledge modeling to address issues raised due to various inconsistencies, e.g., non-standard terminologies. The authors evaluated four different knowledge acquisition scenarios and reported higher classification accuracy for the hybrid approach with formal knowledge verification.

A knowledge based CDSS was proposed by Afzal et al. [42] for treating cancer patients. In this regard, an automated knowledge acquisition approach was proposed to acquire relevant data from head and neck cancer patients’ unstructured documents. Finally, a CART model was used for treatment regimen prediction. The authors reported 69.0% accuracy in correctly selecting the treatment recommendation with respect to routine clinical practice.

Bach et al. [43] proposed a clinical dashboard to facilitate co-decision making in the management of non-specific low back pain patients. The system collects data from questionnaires and wearable devices to make predictions about the course of non-specific low back pain treatment. A case-based approach is used to provide personalized recommendations for patients. The knowledge acquisition and recommendation process are primarily based on pain management guidelines, consultation with clinicians, and past patient cases.

Ali et al. [44] proposed a multi-modal-based interactive authoring environment for expert knowledge acquisition that is also shareable. A case study on oral cavity lesions treatment plan generation was presented in which expert-based knowledge in the form of a mind-map was converted into a set of medical logic modules. In this study, the authors attempted to automate the process for shareable knowledge creation in a user-friendly manner. The proposed system was evaluated from both system-oriented and user-oriented aspects.

Wit et al. [45] evaluated clinical rules in a standalone pharmacy-based CDSS for hospitalized and nursing home patients. The authors investigated the utility of clinical rules for reducing prescription errors. The knowledge acquisition process for creating clinical rules was based on guidelines that are developed by both pharmacists and physicians. The main objective of the study was to evaluate the clinical significance of automated alerts in routine clinical practice. In this regard, the relevance was determined whether or not the pharmaceutics contacted the physician for each alert. The authors reported that the average efficiency of the CDSS was low, whereas a few clinical rules have an efficiency of greater than 10%. A number of factors contributed to the low efficiency of the system, such as alert fatigue and the daily recurrence of previously evaluated alerts, etc.

The aforementioned studies performed knowledge acquisition for developing the expert-driven model. The main focus was on the completeness of the model, i.e., the developed expert knowledge model is sufficient for providing a recommendation for a given task, e.g., treatment selection. The main advantage of the proposed hybridization approach is that it can synthesize partial domain models, i.e., the domain model is used only to provide abstract level generic recommendations. The abstract recommendation is refined using clinical cases that are relatively easy to acquire as compared to rigorously constructing a detailed domain model for complex recommendations such as medication selection and dosing adjustment. Moreover, the proposed approach of synthesizing a partial domain model with clinical cases is also more practical where codified domain knowledge, e.g., clinical practice guidelines, are not sufficiently available for the task at hand. For example, in the domain of CKD–MBD management, the leading guidelines do not provide a detailed recommendation model for dealing with medication selection and dosing prescription. Therefore, general pointers are extracted through domain experts to construct a generic model as per the recommendations of the guideline, while the specialization of the recommendation is aided through the clinical practice of the clinicians in the form of specific cases. Therefore, the main role of the proposed approach is to combine the abstract domain model with that of specific clinical cases for final multi-factor recommendation generation. Table 2 provides a summarized comparison of related techniques for knowledge acquisition for domain model construction.

### 2.2. Medication Selection and Dosing Adjustment

A CDSS for the management of CKD–MBD in patients with ESRD who receive maintenance hemodialysis has the potential to improve different stages of prescription, such as medication initiation, modification, monitoring, or discontinuation [46,47,48]. Furthermore, it is reported that the usage of CDSSs improves overall adherence to clinical practice guidelines and streamlines the decision-making process of clinicians [49]. Vogel et al. [50] compared the effectiveness of an outpatient renal dose adjustment alert through a computerized provider order entry (CPOE) CDSS and a CDSS providing alerts to pharmacists. The authors concluded that both types of CDSS resulted in low rates of potential medication errors. In prescriber-based CDSSs, a pre-defined process map is used to aid in the decision-making of medication prescription and dosing.

Hellden et al. [51] evaluated the impact of a CDSS on the general practitioners’ (GPs) experience of drug dosing. The information-gathering process included a questionnaire and a focus group discussion. The study presented favorable evaluations by GPs in terms of ease of use and overall usefulness in medication dosing. Furthermore, primary care physicians have reported higher acceptance of simple graphical user interfaces, along with task-oriented clear navigation and concise advice.

Pirnejad et al. [52] proposed a methodology for appropriate drug therapy recommendations for kidney transplant patients based on clinical knowledge as well as international recommendations.

Niazkhani et al. [53] proposed a context-aware CDSS for managing drug-laboratory interactions in order to reduce medication errors. The main focus of the study was to develop a user-friendly CDSS to accommodate drug-laboratory interactions (DLIs) while reducing the alert fatigue of clinicians. The knowledge base was based on DLI-rules that were extracted from pharmacology references and clinicians’ direct input. The overall efficacy of the system was evaluated using the “Questionnaire for User Interface Satisfaction”.

Shemeikka et al. [54] proposed a CDSS to support prescriptions of pharmaceutical drugs in patients with reduced renal function. The proposed system was integrated with an electronic health record system (EHR) used in both hospitals and outpatient facilitates. The evaluation of the system was based on a usability questionnaire and the frequency of system logging. The main focus of this research was to integrate the CDSS in the Janus toolbar for appropriate drugs therapy recommendations.

Awdishu et al. [55] proposed a CDSS for supporting medication prescription for CKD patients. The system targeted 20 medications and aided clinicians in the drug therapy discontinuation or dosage adjustment for adult patients with impaired renal function. Medication alterations were based on reviewing primary literature and CPGs, among other resources. The authors reported that the proposed CDSS achieved favorable results in providing guidance on new prescriptions.

Ting et al. [46] proposed a hybrid case-based reasoning approach for medication prescription recommendations. In this regard, the proposed approach combined results from case-based modeling and Bayesian reasoning using a set of heuristic rules. Highly recommended medications were those that were selected by both models. The main focus was on utilizing the clinical experience of physicians along with modeling clinical knowledge in the form of a Bayesian network.

Medication prescription recommendation is a non-trivial task that includes the selection of medication from among a number of alternate medications. Furthermore, medication dosage selection adds to the complexity of the task. For such complex scenarios, clinical practice guidelines (CPGs) do not sufficiently capture the wide range of suitable recommendations. Therefore, most of the studies utilizing domain knowledge can only provide medication selection recommendations. In the proposed hybridization pipeline, we demonstrate an approach which involves combining an abstract domain model with that of clinical cases for medication dosing estimation. In this regard, the proposed approach combines an expert-based model for medication selection and a statistical technique, such as an interquartile range (IQR), for medication dosage estimation. Table 3 provides a summarized comparison of related techniques for medication prescription.

## 3. Methodology

**Figure 3:**
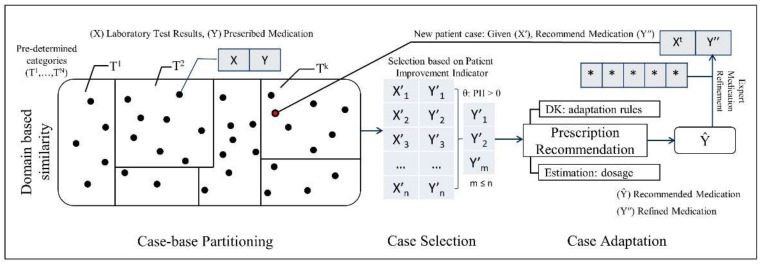
Schematic representation of the proposed hybrid knowledge modeling approach.

The proposed CDSS takes into account the laboratory and imaging test results of patients and assists clinicians in selecting an appropriate treatment regimen. Treatment recommendations in terms of medication selection and dosage adjustment are based on similar patients and domain knowledge. The proposed hybrid approach is illustrated in Figure 3. The expert knowledge is codified into a hierarchical structure such as a DT and it is utilized for partitioning the past clinical cases into multiple groups.

Each case is composed of two components, i.e., problem component and solution component, represented by *X* and *Y*. The problem component represents measurements for multiple laboratory test results such as PTH, phosphate, and albumin-corrected calcium levels along with the status of vascular calcification in the body. The solution component specifies different prescribed medications along with the dosage. A new patient encounter, *X^t^*, is treated as a test case and assigned one of the recommendation groups.

Moreover, each medication recommendation group is denoted by *T^i^*, where *i* refers to the number of pre-specified partitions by the domain experts, and *k* represents any specific partition that contains *X^t^*. In the proposed CKD–MBD CDSS, the entire case base is divided into 33 mutually exclusive partitions, starting from *T*^1^ up to *T*^33^ (refer to Table A1). *X′* and *Y′* represent cases from partition *T^k^* that are treated as similar cases for the given *X^t^*. Moreover, a subset of reference cases, *m*, are selected from the *T^k^* partition containing *n* cases where *m* ≤ *n*. All similar cases are assigned an outcome value based on the PII and only those cases are selected as reference cases that have a PII > 0. Reference cases along with domain knowledge-based adaptation rules are used for prescription recommendation denoted by *Ŷ*. *Ŷ* represents a set of selected medications along with their dosage range, e.g., Medication <Cinacalcet>: = 25 mg/day–50 mg/day. The IQR is used for estimating dosage ranges for multiple selected medications. The prescription recommendation is provided to the clinician that may further refine the dosage. Afterwards, a final prescription, *Y″*, is provided to the new patient case, *X^t^*.

**Figure 4:**
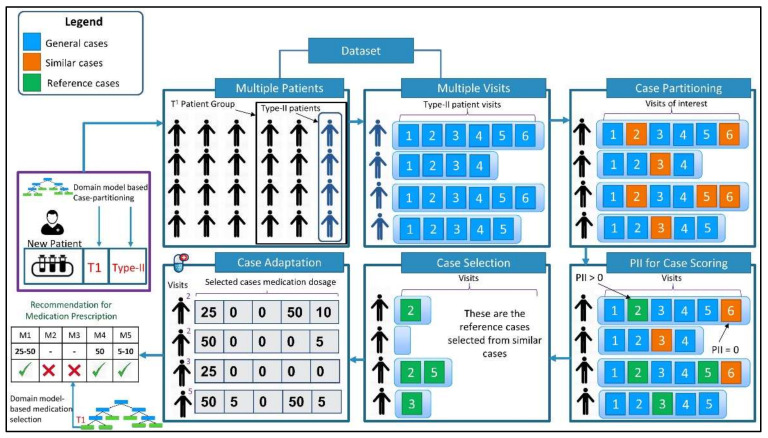
Medication selection and dosage adjustment scenario based on the proposed approach.

A comprehensive recommendation scenario depicting key stages of the proposed recommendation system is depicted in Figure 4. A patient’s laboratory and imaging tests are evaluated through the domain model, and an abstract recommendation is subsequently generated based on the patient’s type, i.e., negative for cardiovascular calcification (type-II), and patient’s group, i.e., T^1^. A set of similar cases are acquired from the case-base pertaining to both type-II and T^1^ patients. Each retrieved case is assigned a case outcome score using the PII. A set of references cases are selected i.e., cases having a PII > 0. Prescribed medications of the selected cases are processed using both adaptation rules acquired from domain knowledge, such as “start or increase medication class A”, “maintain medication class B”, and “decrease or stop medication class C”, etc. IQR is applied on prescribed medication dosages when generating lower and upper bounds, e.g., medication A1: 25 mg/day–50 mg/day, where A1 is one of the medications in medication class A. Adaptation rules are used for medication class level recommendations, i.e., initiation/continuation/discontinuation of a medication class, while clinical cases are used for estimating the lower and upper bounds of dosages for specific medications.

The major advantages of the proposed approach are as follows:

- Domain theory-based patient categorization enhances the confidence of clinicians;
- Each group is denoted by a variable-sized neighborhood;
- Easily identifiable patient groups that have an insufficient number of associated clinical cases;
- Easily identifiable treatment regimens that are effective for similar patient cases;
- Medication dosage adjustment support based on domain theory along with evidence from past clinical cases;
- The enhanced interpretability of the medication selection and dosage adjustment recommendation by clinicians.

**Figure 5:**
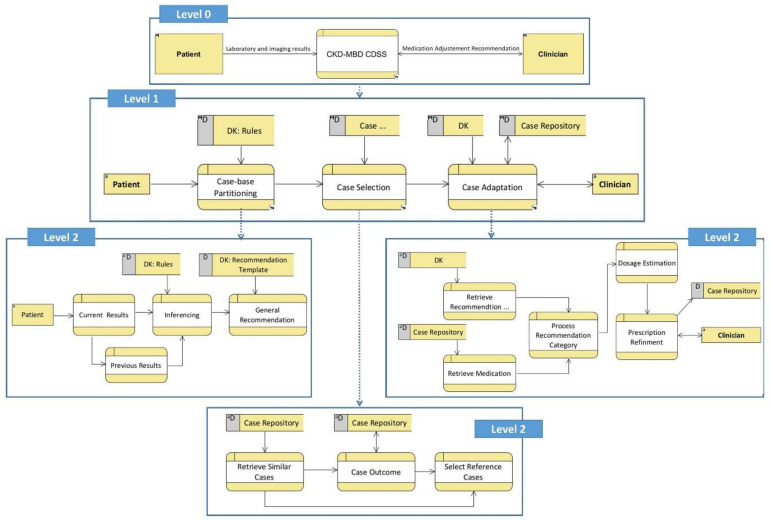
Data flow diagram depicting the relationship between processes and data.

A multi-level data flow diagram (DFD) of the proposed CKD–MBD CDSS is shown in Figure 5. The proposed CDSS is composed of three main tasks, i.e., case-base partitioning, case selection, and case adaptation. An abstract domain model is used to partition the case-base in a pre-determined set of groups, i.e., abstract recommendations. One major advantage of eager partitioning is the active identification of those partitions that lack a sufficient number of clinical cases in the case repository. Case selection deals with retrieving similar cases for a given test case and selecting a set of reference cases from the selected partition. Finally, case adaptation is applied to a set of reference cases with the help of adaptation rules. Adaptation rules are based on domain knowledge. The IQR is used as a measure of statistical dispersion of the selected medication dosages. In this regard, a final recommendation is generated specifying the lower and upper bounds for the dosage values.

### 3.1. Active Case Partitioning through Domain Knowledge

**Figure 6:**
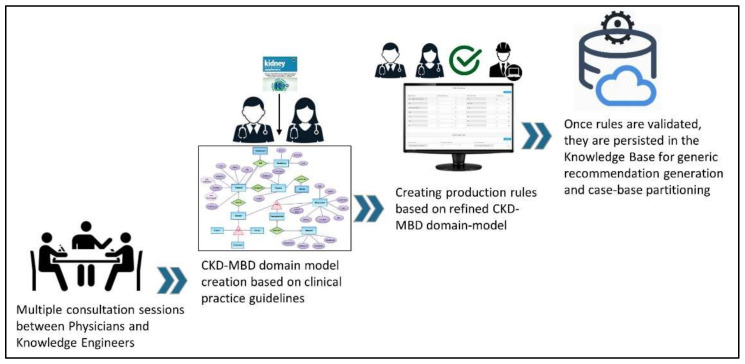
A simplified process for domain-model construction based on clinical practice guidelines for CKD–MBD management.

Domain knowledge (DK) plays a critical role in the development of CDSSs. It primarily deals with specifying key concepts and the relationships among the concepts. In the proposed system, DK is used for a priori partitioning of the case-base into multiple patient groups. The process of DK acquisition and codifying it into a domain model is depicted in Figure 6. DK is also used for generating generic medication intake recommendations that are also used in case adaptation operations for medication selection. The benefits derived from active case partitioning include the variable size of each partition (i.e., neighborhood size is not defined a priori) and, as the relevant cases are localized, this therefore reduces the run-time processing for retrieving similar cases every time a new test case is received.

**Figure 7:**
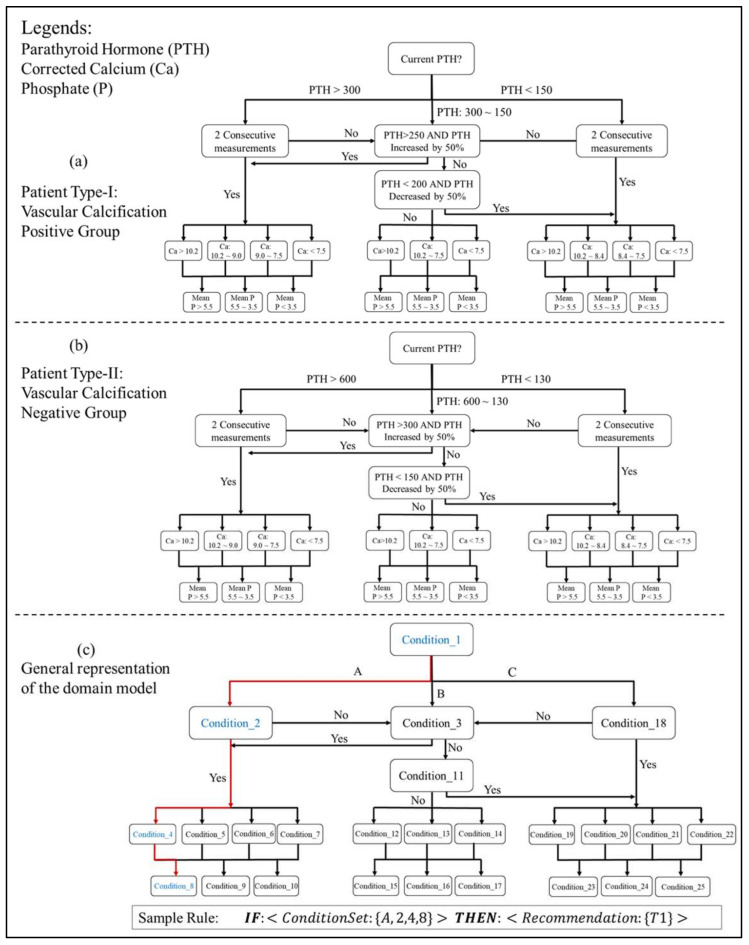
Mind-maps for expert-based domain models for (**a**) type-I and (**b**) type-II CKD–MBD patients along with (**c**) a sample mind-map structure for representing a CPG-based domain-model (Ca refers to albumin-corrected calcium).

The final output of the knowledge acquisition process is a domain-decision model that is similar to a DT structure. Over the course of multiple consultations, the domain experts develop a domain-decision model based for the most part on KDIGO CKD–MBD guidelines [12]. The domain model provides sufficient knowledge to group patient case-base into multiple categories. The domain model is converted into production rules of the form IF-THEN. Figure 7 illustrates a mind-map depicting a domain model for CKD–MBD patients.

As recommended in the KDIGO guidelines, all hemodialysis patients are subject to lateral abdominal radiographs and echocardiography in order to evaluate vascular and valvular calcification, respectively. The severity of vascular calcification is graded on the abdominal aorta by a validated method [56], while valvular calcification is assessed in a dichotomous manner, i.e., its presence or absence.

In the proposed CDSS for CKD–MBD management, hemodialysis patients are broadly categorized into two types based on the degree of ectopic calcification (as shown in Table 4): type-I patients who have valvular calcification or at least a moderate degree of vascular calcification (calcification score > 5 out of 24), and type-II patients who are negative for valvular calcification and have a mild degree of vascular calcification (calcification score ≤5) at most. The novel approach of the proposed CDSS is that a strict target range of PTH is set for type-I patients, whereas a relatively lenient target range of PTH recommended by KDIGO is set for type-II patients. Patient type categorization is performed by domain experts through the consultative method as mentioned in Section 2.1 as a part of the domain knowledge acquisition process.

In this regard, the resultant domain model accommodates both types of patients. There are three key attributes to the domain model, i.e., PTH, albumin-corrected calcium, and phosphate levels in the body (Figure 5). Furthermore, there are in total 33 patient groups identified by the domain experts. Each group is associated with a generic recommendation. A template for the multi-factor generic recommendation is provided in Table 5. ‘Dialysate Calcium Concentration’ is a non-medication factor that can be modified according to the partition to which the patient belongs. The recommendation against each factor is provided in general terms, such as whether to initiate (or increase) a particular medication/dialysate calcium concentration or discontinue (or decrease) the medication/dialysate calcium concentration. Table 6 provides a generic recommendation template. As it can be seen from the table, each factor can take on one of the available treatment options.

Active case partitioning through domain knowledge serves two purposes, i.e., it partitions cases into multiple groups, and it also provides generic medication intake recommendations for each category (refer to Table A1). It is also important to note that one of the main objectives of the domain model construction is to include CKD–MBD guidelines in the decision-making process. The overall domain model for type-I and type-II patients has resulted in 432 production rules (see Figure 7). The production rules are useful in automating the reasoning process and maintaining the knowledge base.

### 3.2. Reference Case Selection Using the PII

**Figure 8:**
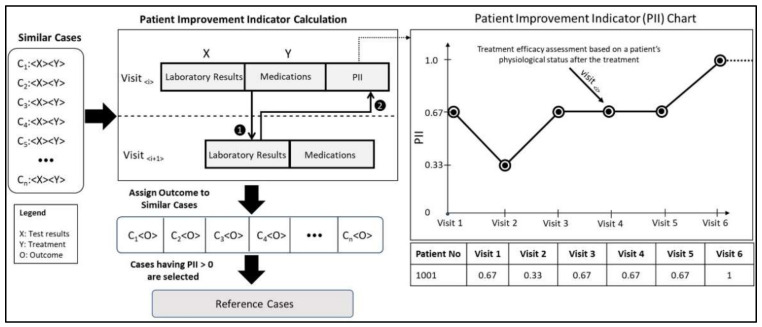
The Patient Improvement Indicator (PII) for selecting reference cases.

One of the important contributions made in this paper is the development of the PII as a case scoring function. The main objective of the PII is to provide a summarized view to the clinician regarding the overall health status of the patient, i.e., patient-important outcome. The PII is comprised of three individual factors i.e., PTH, calcium, and phosphate levels in the body. A patient may visit multiple times over the period of treatment, and at each visit the aforementioned three clinical measurements are used to calculate the PII. The operation of PII computation is depicted in Figure 8 and the formula for its calculation is provided in Equation (1).

(1) $$PII=\frac{\sum_{i}^{m}C_{i}}{m}$$

where, *m* refers to the total number of clinical measurements, *i* refers to the *i*-th measurement, *C* refers to a Boolean value, i.e., either 0 or 1. Each *Ci* value refers to a binary decision, i.e., whether the given test results are within a target range or not. For example, for patients with at least a moderate degree of vascular calcification (i.e., patient type-I), the ideal PTH level is between 150~300 pg/mL [57], while target ranges of phosphate and albumin-corrected calcium are 3.5~5.5 and 7.5~10.2 mg/dL, respectively.

The PII is bounded between 0 and 1. PII values closer to 1 indicate a better patient-important outcome, as depicted in Figure 8. The PII differs for different patients: seeing as the normal range of PTH varies with the type of patient, PII is therefore calculated accordingly.

All the patient cases are assigned their respective PII values, except for corner cases, such as a patient having only a single encounter or the latest encounter of the patient. These cases are treated as corner cases because no subsequent patient encounter is available to calculate the PII value. It is important to note that for evaluating the efficacy of medication dosage prescribed on encounter *i*, the laboratory test results from the subsequent patient encounter, *i* + 1, are required, as shown in Figure 8.

The PII is used to differentiate cases based on their outcome, i.e., whether the patient’s condition (indicated by laboratory test results) improved given a certain prescription or not. The main purpose is to select a set of reference cases that have a positive impact on the outcome, i.e., improvement is recorded in the patient’s laboratory test results. Equation (1) is used to assign an outcome score to a clinical case. An important contribution of the PII is to refine case selection operation, i.e., select cases among similar cases based on their outcome. The refined selection provides a set of reference cases for medication dosage estimation. A similar approach was adopted by Bach et al. [43], whereby the authors initially retrieved a set of similar patients in the domain of low back pain therapy recommendations, and later a reference patient group was identified for further recommendation tuning. The reference group was comprised of patients with positive outcomes, such as decreased pain, improved pain self-efficacy, and better mood. In the case of the CKD–MBD domain, treatment prescription (i.e., medication and dosage selection) for a similar set of patients may vary as per the clinicians’ decisions. The PII is therefore used to qualify different prescribed past treatments according to their efficacy.

### 3.3. Case Adaptation through Domain Knowledge and Clinical Cases

The main objective of the case adaptation operation is to provide medication intake recommendations to the physician. In this regard, domain knowledge and high prospect cases are used for generating recommendations. As stated earlier, domain knowledge is used for both case-base partitioning as well as generic recommendation generation. Case adaptation refines the expert-based generic recommendation through processing similar cases and statistically analyzing the co-occurrence of medication dosages.

**Figure 9:**
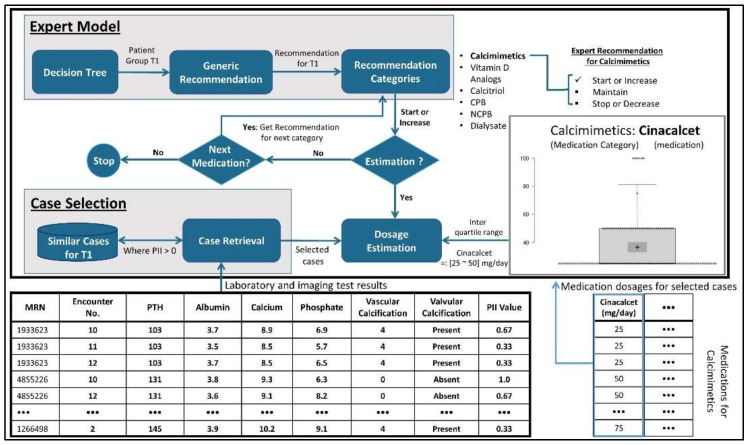
Medication dosage selection and dosage adjustment based on domain knowledge and interquartile range (IQR).

Case adaptation is the final step in the proposed methodology, through which a more precise treatment recommendation is generated that deals with both medication selection and dosage recommendation. Table 7 shows an example of a sample relationship between the generic recommendation and dosage recommendation. As it can be seen that dosage recommendation relies on directions from generic recommendations, i.e., whether to select a particular class of medication or not. Moreover, the final dosage recommendation is based on the most frequent medication and its dosage among high prospect similar cases. It is important to note that, when using IQR statistics, situations in which similar cases take on different medication dosages for a given medicine are reflected in the final recommendation as a dosage range having both lower bound and upper bound values, as shown in Figure 9.

It is important to note that the dosage recommendation is calculated based on the direction from generic recommendations and high prospect similar cases. In this regard, as indicated in Table 7, the generic recommendation for Calcimimetics is “Start or Increase”, Cinacalcet is therefore recommended between 25 mg/day and 50 mg/day. Dosage range estimation is based on IQR of high prospect similar cases.

### 3.4. CKD–MBD CDSS Execution Process

**Figure 10:**
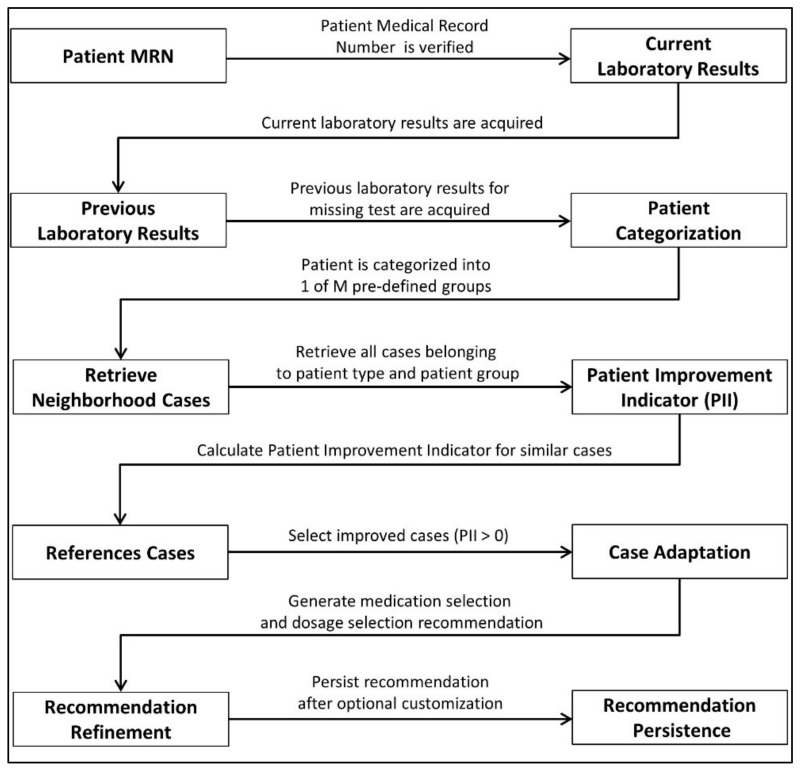
Process flow for CKD–MBD CDSS pertaining to the treatment regimen selection.

The execution process workflow pertaining to the medication prescription is depicted in Figure 10. Patients are assigned a unique Medical Record Number (MRN) at the registration stage. Afterwards, both current and previous laboratory tests are acquired for patient type selection as well as patient group identification (through the domain-decision model). The medical laboratory tests include measurements for phosphate, calcium, albumin, and PTH. A set of reference cases is selected based on the PII of similar cases. Furthermore, if only a single case is available in the selected case set, then it is provided to the physician without any modification. When there are multiple selected cases in the set, then a case adaptation operation takes place that generates a single medication prescription recommendation based on the multiple reference cases. Before persisting with the medication dosage, the clinician may choose to modify the contents of the recommendation, such as adjusting the medication dosage from the recommended one. The system automatically logs the concordance between the generated recommendation and the clinician’s prescription.

The categorization of patients has two aspects, i.e., patient type selection and patient group selection. Patient type selection requires medical imaging results such as lateral abdominal radiography and echocardiography in order to determine the degree of ectopic calcification. The aforementioned imaging tests are conducted once every year. Patients are divided into two types, i.e., positive for vascular calcification and negative for vascular calcification. Patient group selection, on the other hand, is performed using the domain model, as shown in Figure 7. The group selection decision is taken every month, i.e., at each encounter with the patient. Furthermore, PTH laboratory medical results are conducted every three months, and both albumin-corrected calcium and phosphate tests are performed every month. Current and previous laboratory test results are required for patient group selection through the domain model. There are 33 different patient groups identified by clinicians within the scope of CKD–MBD management.

## 4. Experimentation and Results

The CKD–MBD CDSS is evaluated using two perspectives, i.e., system perspective and user perspective. In the case of the system perspective, the evaluation is performed in terms of compliance between the CKD–MBD CDSS medication recommendation and routine clinical practice. The usability aspects of the proposed system are evaluated in terms of recommendation generation, assistance in preventing accidental dosage errors, and serial trend visualization of key measurements such as PTH, phosphate, and albumin-corrected calcium.

### 4.1. System-Centric Evaluation

To validate the system, we performed an experiment in which we established a concordance between the CDSS generated recommendations and that of physician’s prescribed medication. We have 850 clinical cases extracted from 66 patients (each patient had at most 13 encounters) from Kyung Hee University Hospital, Seoul, South Korea. The gender ratio of the patients was 70:30, where 70% of the patients were male. Furthermore, the distribution of clinical cases between type-I and type-II patients was 374 and 476, respectively.

#### 4.1.1. Domain Model Compliance

**Figure 11:**
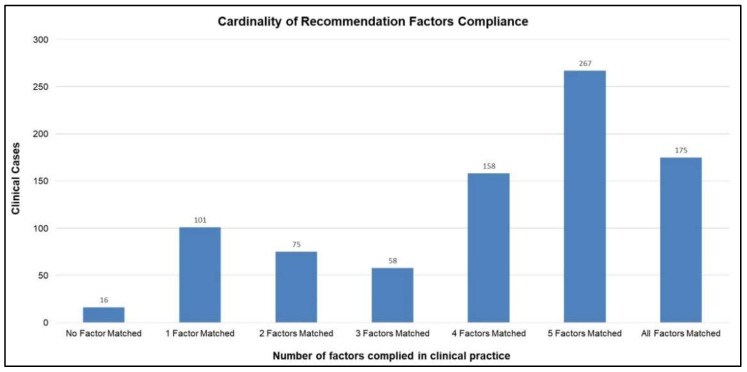
Cardinality of compliance among domain model and routine clinical practice for multi-factor recommendations.

**Figure 12:**
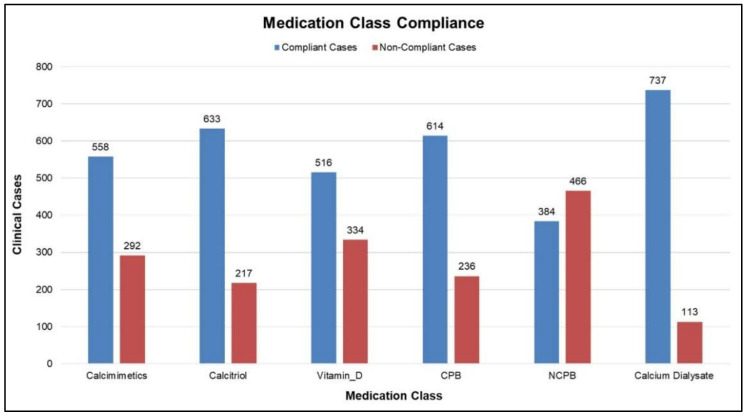
Compliance among different medication management classes along with dialysate calcium concentration.

The domain model is primarily based on KDIGO CKD–MBD guidelines. As mentioned earlier, the generic recommendation is based on the domain model; therefore, it is worthwhile evaluating the compliance between the routine practice and the domain model. The recommendation consists of general directions for clinicians regarding the initiation, modification, or discontinuation of a certain medication class, as indicated in Table 6. The evaluation results, as provided in Figure 11, show the overall compliance between the clinical cases and the generic recommendation. It can be seen in Figure 11 that in general most of the recommendation factors have complied with the routine clinical practice as well. Therefore, the domain model-based generic recommendation plays an important role in the dosage estimation task. Figure 12 shows a breakdown of the compliance rate of six medication classes that are part of the overall recommendation. In the non-compliant cases, “decrease” slightly dominated, e.g., the system recommended to “maintain” while the clinician decreased the dosage.

**Figure 13:**
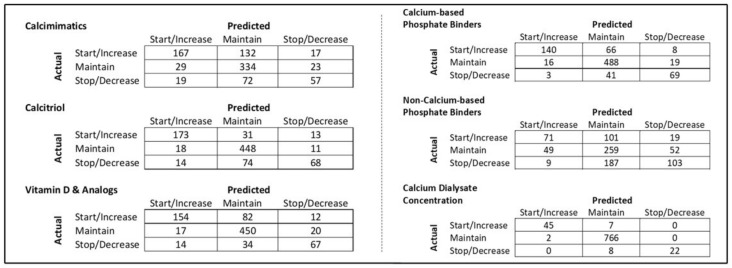
Confusion matrix indicating compliance between domain model and routine clinical practice.

A confusion matrix based on the compliance evaluation between domain model and routine clinical practice is provided in Figure 13. “Maintain” remained the most dominant label in the recommendation across the medication classes. The average discrepancy across all the medication management classes for the “start/increase”, “maintain”, and “stop/decrease” out of 850 cases is 97.50 cases, 42.16 cases, 82.83 cases, respectively. Moreover, NCPB had major discrepancies among all the medication classes, specifically in the “start/increase” recommendation.

#### 4.1.2. Evaluation for Dosage Recommendation

The medication dosage recommendation is the main objective of the CKD–MBD CDSS. In this regard, both domain knowledge and similar past cases are used to assist clinicians in dosage prescription. The clinical case-base consists of 600 cases whereas 250 cases are used to evaluate the recommendation system’s efficacy with respect to the routine clinical practice of clinicians. There are 107 cases for type-I patients and 143 cases for type-II patients in the test dataset. Table 8 provides evaluation results based on the test data, indicating concordance between the dosage recommendation and clinical practice.

(2) $$Concordance=\frac{\sum_{i}^{j}\left(System\;\cap \;Clinician\right)}{j}$$

The evaluation procedure is based on comparing the recommended dosage with that of the clinician’s prescription using Equation (2), where *i* starts from 1 and *j* is the total number of factors in the recommendation, i.e., 10. Seeing as the recommended dosage is based on the IQR, i.e., 1st quartile and 3rd quartile, in most of the cases the recommendation is therefore in the form of a range of values, i.e., lower bound of the dosage and upper bound of the dosage. In such cases, the evaluation is based on whether the prescribed medication dosage is within the recommended dosage range or not. “In-Range” cases are those in which the prescribed medication is within the recommended range; otherwise they are regarded as “Out-of-Range” cases. Furthermore, not all medications are present in all the cases, i.e., Cinacalcet is present in 49 cases out of a total of 250 test cases, and so on. Concordance for Cinacalcet is 85.71%, Calcitriol (po) is 81.81%, Calcitriol (iv) is 66.66%, Paricalcitol (iv) is 82.24%, Calcium Carbonate is 76.47%, Calcium Acetate is 81.81%, Sevelamer is 76.12%, Lanthanum is 55%, and Dialysate calcium concentration is 98.40%. As Alfacalcidol does not include any case in the test set, it is therefore not part of the average concordance calculation. The average concordance of the medication dosage recommendation, as reported in Table 8, is 78.27%.

### 4.2. User-Centric Evaluation

The usability of the system is yet another important consideration apart from its efficacy. Systems that have bad user experiences, such as unnecessary complexity, workflow inconsistency, and distraction, lead to cognitive burdens on the user and results in limited usability. In this paper, we have also evaluate the usability aspect of the proposed system.

The system is evaluated by 11 participants with different experience levels and expertise with healthcare applications. The system is evaluated in an end-to-end manner including tasks such as patient registration to the final recommendation generation and prescription persistence.

The CDSS features under evaluation include user interfaces for recommendation generation, consistency of the user interfaces, timeliness of the relevant information, visualization of the clinical parameters, medication dosage-related pop-ups, among others.

Participants’ responses are acquired through a widely popular user experience evaluation questionnaire. Questionnaires are widely used as a research instrument for effective user experience evaluation. The User Experience Questionnaire (UEQ) compares the level of experience and assessed scale means of participants with a benchmark dataset of 4818 people across 163 studies on various services [43].

**Figure 14:**
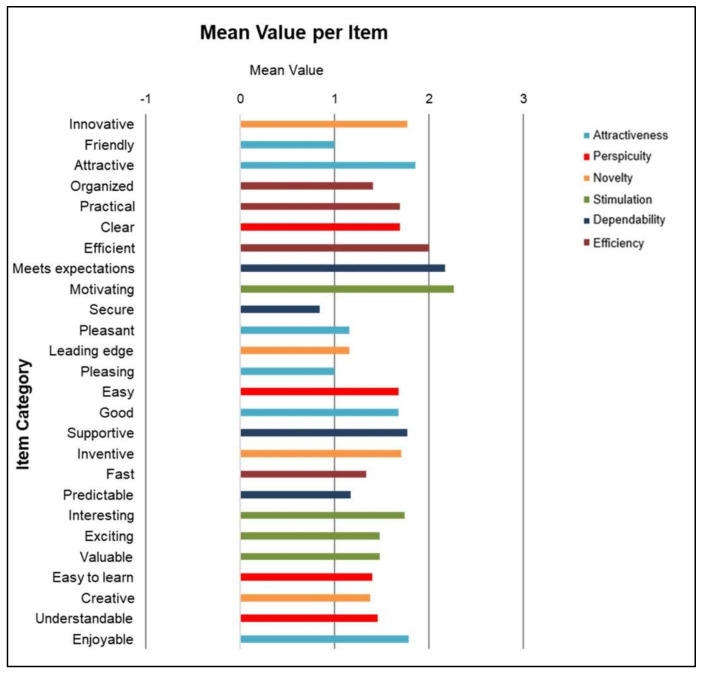
Scale mean value per item for multi-aspect user experience (UX) evaluation.

Figure 14 lists a number of key items describing a distinct quality aspect of an interactive product identified by usability experts. UEQ contains six user experience (UX) aspect scales with 26 items. Items belonging to a specific group are similar in meaning but represent different aspects of the system for a given aspect scale. The Cronbach’s alpha coefficient is a well-known metric for determining the average value per item [58]. Figure 14 demonstrates that the 50 percent mean values are more than or equal to 1.5, confirming the proposed system’s substantial positive impact on the UX of the participants.

The overall six scales are attractiveness, perspicuity, efficiency, stimulation, and novelty. In this regard, attractiveness is a pure valence dimension. Furthermore, perspicuity, efficiency, and dependability are pragmatic quality aspects (goal-directed), while stimulation and novelty are hedonic quality aspects (not goal-directed). Attractiveness represents an overall impression of the system. Perspicuity characterizes ease of familiarity with the system, efficiency represents whether users can solve their task without unnecessary effort. The dependability aspect denotes if user feels in control of the interaction. Stimulation represents whether it is an exciting and motivating product to use or not. And finally, novelty characterizes whether the system catches the interest of users or not?

**Figure 15:**
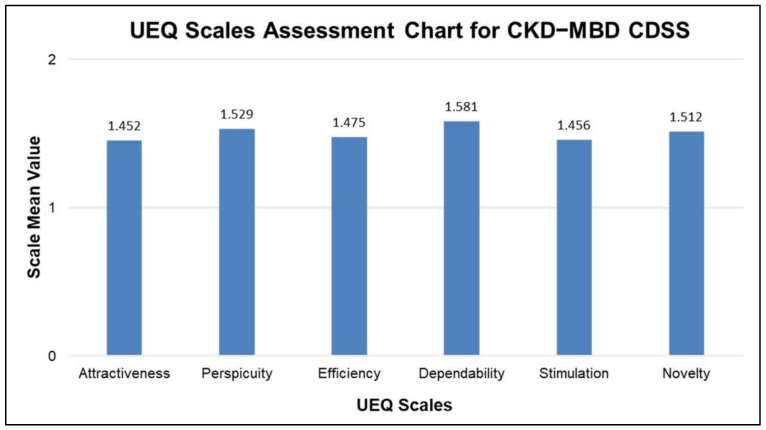
User Experience Questionnaire (UEQ) scale values for key 6 aspect dimensions.

As shown in Figure 15, the analysis of UEQ support is used to determine the means of stimulation, attractiveness, perspicuity, dependability, efficiency, and novelty scales [58,59] in the 0 to 2 range. The value of the dependability scale is close to 2.0, [60] indicating that the proposed system induces confidence in the decision-making of the participants.

The 95% confidence intervals for the UEQ scale mean are used to evaluate the confidence interval (a measure of the precision of mean estimation) [61]. The mean confidence scores calculated are 1.452, 1.529, 1.475, 1.581, 1.456, 1.512 for attractiveness, perspicuity, efficiency, dependability, stimulation, and novelty, respectively, as shown in Figure 15.

**Figure 16:**
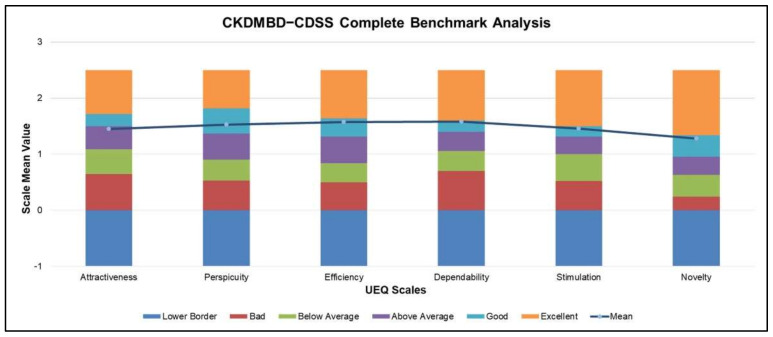
CKD–MBD CDSS Benchmark Analysis.

The UEQ tool compares the UX of the proposed system with that of other services [61]. As indicated in Figure 16, the system provides higher dependability due to the transparency of its decision-making along with the inculcating of domain knowledge. Moreover, other aspects such as perspicuity, efficiency, and stimulation are also in the “Good” range, indicating a general acceptance across the participants. The attractiveness aspect of the system is “Above Average”, while the novelty aspect is also reasonably high, indicating participants’ interest in using the system [62].

## 5. Discussion

**Figure 17:**
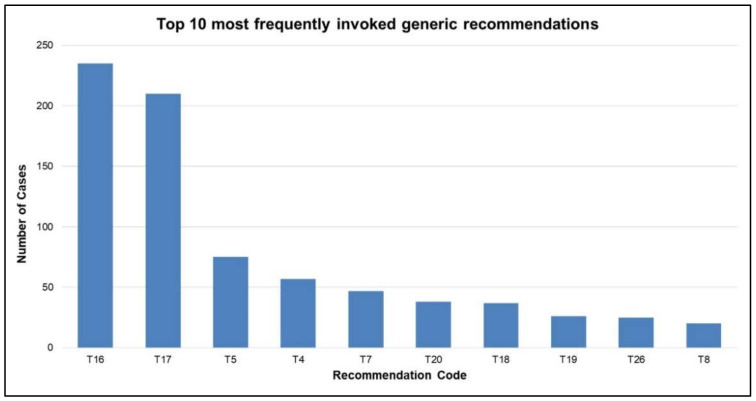
Top 10 frequent generic recommendations.

The domain model plays a critical role in the proposed methodology as it guides the case adaptation operation along with providing it with a subset of relevant cases for estimating the medication dosage. The compliance between the clinical practice and the domain model depends on the overall compliance rate, which is generally high due to the fact that some of the highly frequent recommendations have a high compliance rate. In this regard, it is observed that among 33 different generic recommendations, only a few of the recommendations are more frequent, as shown in Figure 17. The medication dosage is mostly kept consistent, avoiding abrupt changes from one encounter to another, whichcorroborates long-term treatment regimens. Therefore, both T16 and T17 recommend to “maintain” the medication dosage in general, while suggesting little changes in the medication selection and dosing. The aforementioned observation also explains the relatively higher frequency of these two recommendations.

The striking gap between real-world practice and algorithm-directed recommendation lies in non-calcium-based phosphate binders (NCPB). In most cases, physicians did not increase the dosage of NCPB despite elevated serum phosphate levels. The side effects of NCPB, which frequently causes nausea, vomiting, and abdominal discomfort, may be behind this lack of increases in dosage. It could also be the case thay physicians were likely to be reluctant to actively prescribe NCPB due to pill burden, since more than six tablets a day are required to meet recommendations in some cases. Our results reflect the practical difficulties of lowering elevated phosphate levels which are encountered by most physicians. Moreover, in some cases, the serum phosphate levels remained slightly higher than the upper limit of the target range, prompting the system to increase NCPB dosage while the clinician opted to maintain the current dosage. This behavior is due to the crisp nature of the rule-base, with little tolerance for on-the-edge cases.

As recommended by the KDIGO guidelines, dialysate calcium concentration in most cases was 1.25 mmol/L, and “maintain” was the most frequent recommendation. Since dialysate calcium concentration was usually unchanged, the overall compliance rate was the highest. In a few exceptional cases of severely low blood calcium levels, “increase” was provided as a last resort.

In the case of medication dosage recommendations, a high level of concordance is found for Cinacalcet. This can be explained by Cinacalcet being a single medication option available for prescription in the class of Calcimimetics within the scope of the proposed system. In all other medication classes, there are at least two medication options available, e.g., both Sevelamer and Lanthanum fall under the medication class of NCPB, whereas the CPB medication class includes medications such as Calcium Carbonate and Calcium Acetate.

In terms of user-centric evaluation, the proposed system obtained a high score on dependability. This can be attributed to several reasons, such as adopting domain knowledge in decision making, indicating the selected relevant cases, quantifying patient-important outcomes in the form of the PII, and evaluating the system with real-world patient data. The perspicuity aspect of the system, on the other hand, is also underscored by these results, as the system is user-friendly to navigate and the required information for decision making is readily available. The attractiveness of the system can be enhanced by improving the user interfaces (UI), such as de-cluttering the UI elements from the recommendation panel and demarking clear boundaries when multiple information panels are displayed in close proximity, such as in the cases of laboratory test results, prescribed medications, and recommended medications.

In terms of limitations, the proposed system heavily relies on domain knowledge. Acquiring accurate and consensus-based knowledge from domain experts would therefore pose a challenge when adopting the proposed methodology where CPGs are not readily available. Furthermore, in the context medication dosage estimation, all the pre-defined partitions must have member cases associated with them. In the case of a partition that does not contain any clinical case, the dosage estimation operation cannot be efficiently performed.

## 6. Conclusions

CDSSs assist clinicians and healthcare providers in both complex decision-making and addressing routine healthcare tasks. CDSSs process and analyze healthcare data, e.g., laboratory and imaging test results, in addition to medication history in order to provide prompts and reminders at the point of care. Applied to CKD–MBD management, CDSSs can assist clinicians in the selection of appropriate treatment protocols and tailored recommendations based on the status of vascular calcification.

In this study, a hybrid knowledge modeling approach is proposed that incorporates both domain knowledge and patients’ clinical cases for complex decision making, such as appropriate initiation, modification, monitoring, or discontinuation of the medication. Furthermore, we propose a PII which provides an overall summary of the patient record over a period of time. The PII is helpful in identifying past similar cases that have positive patient-important outcomes, e.g., patient laboratory tests that have improved with the prescribed medication regimen, so that similar patients may also be recommended the same medication regimens. Medication dosage estimation is performed on reference cases (acquired from similar patient cases) using the IQR to assist clinicians in selecting appropriate dosing.

The proposed system is evaluated based on 250 clinical cases from hemodialysis patients and the overall concordance is found at 78.27% between the system-provided recommendations and the routine clinical practice. A widely used user experience evaluation tool, UEQ, is used to evaluate the proposed systems’ usability aspects with respect to clinicians. The usability assessment is based on clinicians who have independently evaluated the system. The dependability and perspicuity of the system scored highly, while its attractiveness remained relatively low across the participants. This shows that the system provides useful recommendations along with initiative workflows that seamlessly align with clinical workflows, whereas information displaying panels can be further improved to de-clutter the user interface.

We intend to expand the automated decision-making framework to other comorbidities of CKD–MBD, such as cardiovascular disease, osteoporosis, diabetes, among others. Moreover, patient data from multiple medical centers will be acquired to reflect sufficient diversity of different treatment approaches adopted by clinicians. Bayesian reasoning along with deep learning approaches are some of the candidate approaches that will be evaluated for hybridization along with domain knowledge.

## Figures and Tables

**Table 1:** A list of abbreviations used in this paper.

Abbreviations	Full Form
CKD–MBD	Chronic Kidney Disease-MineralBone Disorder
ESRD	End-Stage Renal Disease
CDSS	Clinical Decision Support System
PTH	Parathyroid Hormone
Ca	Calcium
P	Phosphate
CPB	Calcium-based Phosphate Binder
NCPB	Non-Calcium-based Phosphate Binder
UX	User Experience
UEQ	User Experience Questionnaire
DK	Domain Knowledge
CPG	Clinical Practice Guidelines
UI	User Interface
CART	Classification and Regression Tree
CPOE	Computerized Provider Order Entry
GP	General Practitioner
DLI	Drug Laboratory Interactions
EHR	Electronic Health Records
DT	Decision Tree
IQR	Interquartile Range
MRN	Medical Record Number
ATT	Attractiveness
PQ	Pragmatic Quality
HQ	Hedonic Quality
PII	Patient Improvement Indicator

**Table 2:** Literature Comparison–Knowledge Acquisition.

Reference	Area of Application	Objective	Characteristics	Limitations
[39]	Thyroid nodules	Treatment	Knowledge-based system modeling Expert driven domain model Retrospective evaluation	Complete knowledge model is difficult to acquire Model evolution requires domain expert involvement
[40]	Heart disease	Diagnosis	Hybrid knowledge model Interpretable decision making Retrospective and pilot study	Difficult to express domain consensus for complex cases Combined model is prone to overfitting
[41]	Oral cavity cancer	Diagnosis	Hybrid knowledge model Model consistency evaluation through formal methods Retrospective evaluation	Complete domain model is difficult to acquire for complex decision tasks Combined model is prone to overfitting
[42]	Head and neck cancer	Diagnosis	Automated knowledge acquisition from documents Interpretable decision model Offline and online evaluation	Domain expert involvement is required for data quality validation Resulting model does not incorporate domain knowledge that is not reflected in selected data
[43]	Low back pain	Treatment	Co-decision making model Implicit knowledge modeling through case-based framework Reference group selection based on positive outcome	Clinical guidelines are not integral part of the case-based model Data acquisition through wearable devices is unreliable, and self-reporting data are subjective
[44]	General healthcare	Wellness management	Framework for domain model enrichment Wellness concept model for health management Model evaluated using nominal group technique	Only SNOMED CT is used for standard terminology harmonization Model evolution requires domain expert involvement
[45]	Standard medical care	Treatment	CDSS based on clinical rules for pharmacy application Automated alerts for prescription error reduction Retrospective evaluation	Difficult to express domain consensus for complex cases Model evolution requires domain expert involvement

**Table 3:** Literature Comparison-Medication Prescription.

Reference	Area of Application	Objective	Characteristics	Limitations
[50]	Kidney disease	Medication selection	CDSS based on pre-defined process map Outpatient renal dose adjustment using CDSS Retrospective evaluation	Difficult to express domain consensus for complex cases Model maintenance requires domain expert involvement
[51]	Primary healthcare	Medication selection	A two-step drug recommendation through CDSS Integrated into Janus web solution Evaluation through questionnaire responses and focus group	Complete domain model is difficult to acquire for complex decision tasks with multiple preferences Clinical experience of different clinicians for dosing recommendation is not integral part of the CDSS
[52]	Kidney patients	Medication selection	CDSS for potential drug–drug interactions (pDDIs) recommendation Knowledge base construction for pDDIs alert generation Prospective evaluation	The knowledge does not provide medication dosing recommendation Difficult to express domain consensus for complex cases
[53]	Kidney patients	Medication selection	Context-aware CDSS for drug–laboratory interactions (DLIs) Knowledge base for DLIs recommendations Prospective cross-sectional evaluation using real clinical patient data	Complete domain model is difficult to acquire for complex decision tasks with multiple preferences Difficult to maintain complex rule-based models, e.g., medication adjustment
[54]	Kidney patients	Medication selection	Drug prescription for reduced renal function patients CDSS is integrated in Janus toolbar Evaluation using questionnaire technique	Clinical experience of different clinicians for dosing recommendation is not integral part of the CDSS Clinical experience of medication selection is not reflected in the model
[55]	Kidney patients	Medication selection	CDSS for drug therapy selection/discontinuation Different alerts are designed based on multiple domain sources Prospective evaluation using randomized control trial	The CDSS does not provide medication dosing recommendation Knowledge maintenance for new medications would pose a major challenge
[46]	Standardmedical care	Medication selection	Data-driven hybrid model using case-based reasoning and Bayesian reasoning, execute in parallel Heuristic rules to combine results from both models	The CDSS does not provide medication dosing recommendation Complete domain model is difficult to acquire for complex decision tasks reflecting multiple preferences

**Table 4:** Relevant clinical parameters and their target ranges.

Clinical Parameter	Target Range
PTH (type-I patient)	150~300 pg/mL
PTH (type-II patient)	130~600 pg/mL
Phosphate	3.5~5.5 mg/dL
Albumin-corrected Calcium	7.5~10.2 mg/dL

**Table 5:** A sample generic recommendation.

Management Class	Treatment Options
Calcimimetics	Start or Increase Cinacalcet
Calcitriol	Stop Calcitriol
Vitamin D and Analogs	Stop Vitamin D and Analogs
Calcium-based Phosphate Binder	Stop CPB
Non-Calcium-based Phosphate Binder	Start or Increase NCPB
Dialysate calcium concentration	Reduce by 0.25 mmol/L

**Table 6:** Generic Recommendation Template.

Management Class	Available Treatment Options
Calcimimetics	[Start or Increase Cinacalcet], [Decrease Cinacalcet], [Stop or Decrease Cinacalcet], [As it is]
Calcitriol	[Start or Increase Calcitriol], [Stop Calcitriol], [Decrease or Stop Calcitriol], [Consider Calcitriol], [As it is]
Vitamin D and Analogs	[Consider Vitamin D Analogs], [Decrease or Stop Vitamin D Analogs], [As it is]
Calcium-basedPhosphate Binder	[Start or Increase CPB], [Stop CPB], [Decrease or Stop CPB], [As it is]
Non-Calcium-basedPhosphate Binder	[Start or Increase NCPB], [Stop NCPB], [Decrease or Stop NCPB], [As it is]
Dialysate CalciumConcentration	[Increase by 0.25 mmol/L], [Reduce by 0.25 mmol/L], [Maintain Current Calcium Concentration]

**Table 7:** A sample medication dosage recommendation with respect to generic recommendation.

Generic Recommendation	Dosage Recommendation	Reference Dosage Range
Calcimimetics: Start or Increase	Cinacalcet: 25 mg/day–50 mg/day	Cinacalcet: 0~100 mg/day
Calcitriol: Stop Calcitriol	Calcitriol, po: 0 ug/day	Calcitriol, po: 0~2.0 ug/day Calcitriol, iv: 0~10 ug/week
Vitamin D and Analogs: Stop Vitamin D and Analogs	Paricalcitol, iv: 0 ug/week Alfacalcidol: 0 ug/day	Paricalcitrol, iv: 0~50 ug/week Alfacalcidol: 0~3 ug/day
Calcium-based Phosphate Binder: Stop CPB	Calcium Carbonate: 0 mg/day Calcium Acetate: 0 mg/day	Calcium Carbonate: 0~3750 mg/day Calcium Acetate: 0~6000 mg/day
Non-Calcium-based Phosphate Binder: Start or Increase NCPB	Sevelamer: 800 mg/day Lanthanum: 0 mg/da	Sevelamer: 0~13,000 mg/day Lanthanum: 0~3750 mg/day
Dialysate Calcium Concentration: Maintain current dialysate calcium concentration	Dialysate Calcium Concentration: 1.25 mmol/L	Dialysate Calcium Concentration: 1.25~1.75 mmol/L

**Table 8:** Concordance evaluation for the medication dosage recommendation.

Management Class	Total Cases	Present Cases	In-Range Cases	Out-of-Range Cases
^†^ Cinacalcet	250	49	42	7
Calcitriol, po	250	11	9	2
Calcitriol, iv	250	15	10	5
Paricalcitol, iv	250	148	122	26
^†^ Alfacalcidol	250	0	0	0
^†^ Calcium Carbonate	250	34	26	8
^†^ Calcium Acetate	250	11	9	2
^†^ Sevelamer	250	155	118	37
^†^ Lanthanum	250	20	11	9
Dialysate Calcium Concentration	250	250	246	4

^†^ Cinacalcet, alfacalcidol, calcium carbonate, calcium acetate, sevelamer, and lanthanum are orally taken tablets.

## Data Availability

CKD-MBD prescription data used in this study are available on a reasonable request.

## Appendix A

Table A1; Complete list of 33 mutually exclusive partitions (generic recommendations).

**Table A1:** A list of 33 generic recommendations.

Rcode	Calcimimetics	Calcitriol	Vitamin D and Analogs	CPB	NCPB	Dialysate Calcium Concentration
T1	Start or Increase	Stop	Stop	Stop	Start or Increase	Reduce by 0.25 mmol/L, If more than 1.5 mmol/L
T2	Start or Increase	Stop	Stop	Stop	As it is	Reduce by 0.25 mmol/L If more than 1.5 mmol/L
T3	Start or Increase	Stop	Consider Vitamin D and Analogs	Stop	Decrease or Stop	Reduce by 0.25 mmol/L If more than 1.5 mmol/L
T4	Start or Increase	Stop	Consider Vitamin D and Analogs	Stop	Start or Increase	Maintain current dialysate calcium concentration
T5	Start or Increase	As it is	Consider Vitamin D and Analogs	As it is	As it is	Maintain current dialysate calcium concentration
T6	Start or Increase	As it is	Consider Vitamin D and Analogs	Stop	Decrease or Stop	Maintain current dialysate calcium concentration
T7	As it is	As it is	Consider Vitamin D and Analogs	As it is	Start or Increase	Maintain current dialysate calcium concentration
T8	As it is	Consider Calcitriol	Consider Vitamin D and Analogs	As it is	As it is	Maintain current dialysate calcium concentration
T9	As it is	Consider Calcitriol	Consider Vitamin D and Analogs	Decrease or Stop	Decrease or Stop	Maintain current dialysate calcium concentration
T10	Decrease	Consider Calcitriol	Consider Vitamin D and Analogs	Start or Increase	As it is	Increase by 0.25 mmol/L
T11	Decrease	Start or Increase	Consider Vitamin D and Analogs	Start or Increase	As it is	Increase by 0.25 mmol/L
T12	Decrease	Start or Increase	Consider Vitamin D and Analogs	As it is	Decrease or Stop	Increase by 0.25 mmol/L
T13	As it is	Stop	Stop Vitamin Dand Analogs	Stop	Start or Increase	Reduce by 0.25 mmol/L If more than 1.5 mmol/L
T14	As it is	Stop	Stop Vitamin D and Analogs	Stop	As it is	Reduce by 0.25 mmol/L If more than 1.5 mmol/L
T15	As it is	Stop	Stop Vitamin D and Analogs	Stop	Decrease	Reduce by 0.25 mmol/L If more than 1.5 mmol/L
T16	As it is	As it is	As it is	As it is	Start or Increase	Maintain current dialysate calcium concentration
T17	As it is	As it is	As it is	As it is	As it is	Maintain current dialysate calcium concentration
T18	Stop or Decrease	As it is	As it is	As it is	Decrease	Maintain current dialysate calcium concentration
T19	Stop or Decrease	As it is	As it is	Start or Increase	Start or Increase	Increase by 0.25 mmol/L
T20	Stop or Decrease	Start or Increase	As it is	Start or Increase	Decrease or Stop	Increase by 0.25 mmol/L
T21	Stop or Decrease	Start or Increase	As it is	As it is	Stop	Increase by 0.25 mmol/L
T22	Decrease or Stop	Stop	Stop	Stop	Start or Increase	Reduce by 0.25 mmol/L If more than 1.5 mmol/L
T23	Decrease or Stop	Stop	Stop	Stop	As it is	Reduce by 0.25 mmol/L If more than 1.5 mmol/L
T24	Decrease or Stop	Stop	Stop	Stop	Decrease	Reduce by 0.25 mmol/L If more than 1.5 mmol/L
T25	Decrease or Stop	Decrease or Stop	Decrease or Stop	Stop	Start or Increase	Maintain current dialysate calcium concentration
T26	Decrease or Stop	As it is	Decrease or Stop	As it is	Decrease or Stop	Maintain current dialysate calcium concentration
T27	Decrease or Stop	As it is	Decrease or Stop	Stop	Decrease or Stop	Maintain current dialysate calcium concentration
T28	Decrease or Stop	As it is	Decrease or Stop	As it is	Start or Increase	Maintain current dialysate calcium concentration
T29	Decrease or Stop	Decrease or Stop	Decrease or Stop	As it is	As it is	Maintain current dialysate calcium concentration
T30	Decrease or Stop	As it is	Decrease or Stop	Stop	Decrease or Stop	Maintain current dialysate calcium concentration
T31	Decrease or Stop	Decrease or Stop	Decrease or Stop	Start or Increase	Start or Increase	Increase by 0.25 mmol/L
T32	Decrease or Stop	Decrease or Stop	Decrease or Stop	Start or Increase		Increase by 0.25 mmol/L
T33	Decrease or Stop	Start or Increase	Decrease or Stop	Decrease or Stop	Decrease or Stop	Increase by 0.25 mmol/L
